# A flying ad-hoc network dataset for early time series classification of grey hole attacks

**DOI:** 10.1038/s41597-025-05560-1

**Published:** 2025-08-15

**Authors:** Charles Hutchins, Leonardo Aniello, Enrico Gerding, Basel Halak

**Affiliations:** University of Southampton, School of Electronics and Computer Science, Southampton, SO17 1BJ UK

**Keywords:** Computer science, Electrical and electronic engineering

## Abstract

Flying ad-hoc networks (FANETs) consist of multiple unmanned aerial vehicles (UAVs) that rely on multi-hop routes for communication. These routes are particularly susceptible to grey hole attacks, necessitating swift and accurate defence to preserve the network’s quality of service. Grey hole attacks are a type of denial-of-service attack where malicious nodes selectively drop packets, disrupting the normal flow of data in the network. This paper introduces and motivates a novel dataset, FAN-GHETS24, designed for the fast classification of various grey hole attacks. The dataset is derived from sequences of packet interactions between UAVs within the network, generated through multiple simulations of FANETs. These sequences undergo post-processing via two methods: firstly, an anonymization procedure that replaces IP addresses with standard string variables, allowing for offline model training and deployment on any UAV; and secondly, the application of feature engineering techniques to format the data for machine learning model integration. The dataset’s utility is validated using a time series classification model which focuses on classifying grey hole attacks as quickly as possible.

## Background & Summary

Flying Ad hoc networks (FANETs) are collections of WiFi capable Unmanned Aerial Vehicles (UAVs) or nodes which autonomously build communication routes to provide network services to areas lacking fixed infrastructure. Subsequently, they are used by search and rescue (SAR) teams to provide assistance to victims overcome by natural disasters^1,2^. In these scenarios it is essential that these search and rescue teams provide a timely response to save human lives.

Unfortunately, the protocols which provide these crucial communication links are vulnerable to attack from malicious drones. These routing based attacks include black hole attacks, worm hole attacks, rushing attacks, flooding attacks and modification attacks. The details of these specific threats can be found in a recent survey paper^3^. For the purposes of this article, we focus on another type of routing based attack called a grey hole attack (GHA). GHAs reduce the quality of service (QoS) of the network by posing as legitimate nodes and dropping packets when asked to act as a router. Worryingly, GHAs can be executed in various forms and intensities, and yet, only a few studies have thoroughly investigated these aspects^4,5^. We prioritize the identification of GHAs due to the significant challenges it presents in detection^6,7^.

Due to the threat of GHAs in FANET environments, various countermeasures have been developed^3,7–12^. Categorisation of these countermeasures is extremely difficult, but we provide a primitive categorisation strategy based on three factors: function, response time, and training scheme, to help explain the current trends in the security of FANET networks.

Firstly, countermeasures can be categorized based on their function as either an *intrusion prevention system* (IPS) or *intrusion detection system* (IDS). Typically, IDSs provide classification-style metrics such as F1 score and seek to identify malicious nodes or packet flows. On the other hand, IPSs typically report network performance metrics such as packet delivery rate (PDR) or latency to prove that the system can effectively respond to an attack. Secondly, we define the response time of these systems as ether *real-time* or *delayed* to distinguish between works which actively prevent threats during routing and works which delay their decision making until after the event has happened. Thirdly, the training scheme of a system can either be *online* or *offline*. Specifically, online training schemes develop a model of the network by observing current behaviour. Contrastingly, offline training schemes use previously developed models and apply them to the current situation.

One recent study^8^, proposes a delayed IDS which learns in an offline manner. This work uses network performance statistics such as average round trip time, packet loss percentage and the number of packets received to identify grey hole nodes, black hole nodes, selfish nodes and benign nodes. Furthermore, a variety of machine learning models were trained on this data, including a random forest classifier and a support vector machine. Another study^9^ uses a federated learning based approach to classify worm holes, black holes and flooding attacks. The features used in this study include routing table information as well as metrics related to the number of AODV control packets. By utilising a federated learning based approach, models can be trained on individual UAVs with the weights uploaded to a central server for redistribution to other UAVs. We class this IDS as offline and delayed due to the fact that the features need to be labelled before the UAVs can train on this data.

More advanced machine learning techniques have also been proposed as a counter to grey hole threats. For example, the RORQ protocol^10^ adapts the Q-routing algorithm^13^ to include energy, reputation and current buffer size within the state space. Consequently, this enables the nodes to choose an optimal next hope node based on favourable state information, thereby avoiding GHA nodes. We classify this model as an online real-time IPS. In a similar fashion, another online real-time IPS^11^ also uses reinforcement learning (RL) to optimise the security of FANETs, albeit with different state information. A more recent study^12^ proposes another RL-based protocol which, in contrast, is classified as an offline real-time IPS, eliminating the need for on-device training.

In this article, we focus on the development of offline FANET IDSs which optimise for both fast threat detection as well as accurate classification. These IDSs are partly real-time, as they have the ability to identify nascent threats, but rely on some amount of incoming data before a decision is made. This contrasts sharply with delayed IDSs^8,9^ which need a fixed window of information to make their decision. Specific application areas such as SAR necessitate fast classification of threats to protect vital systems. Surprisingly, we did not find any offline IDS systems which catered to this SAR requirement.

This need for fast classification led us to a body of literature called *early time series classification* (ETSC) which has been successfully applied in other safety critical systems, such as gas leak detection^14^ and blood purification treatment^15^. In addition, the importance of accuracy and earliness is also evident in the cyber security field. Specifically, fast classification models are utilised in malware identification by analysing application programming interface (API) call sequences^16^ and in the hardware security domain^17^ by analysing hardware performance counters.

Training and evaluating an ETSC model for GHA detection in FANETs requires a suitable dataset. Consequently, we have identified 3 essential properties that must be present to ensure its versatility compared with other datasets in the field. Firstly, the dataset needs to be generated from a suitable simulation environment. Secondly, datasets for ETSC models require a slightly different construction compared with other datasets. And thirdly, this dataset must include different types of grey hole attacks to ensure that the trained models can successfully identify all GHA types. We describe these requirements in more detail in the following paragraphs.

The dataset needs to be generated from a platform which correctly simulates FANETs and the affects of GHA nodes on the FANET. There are several prominent datasets in the network security domain, which include the KDDcup’99^18^, NSL-KDD^19^, and the CIC-IDS2017^20^ datasets. These datasets are still used to develop IDS models today^21–23^, however, they are of limited use in FANET environments due to their network configuration. The KDDcup’99^18^, NSL-KDD^19^ and CIC-IDS2017^20^ datasets are generated using simulations of local area networks (LANs) where routing is accomplished through dedicated switches and routers. In contrast, FANETs use a type of ad-hoc networking where the nodes of the network can simultaneously function as senders, receivers, and routers. In addition, the KDDcup’99^18^, NSL-KDD^19^ and CIC-IDS2017^20^ datasets do not incorporate node mobility, a crucial factor influencing the performance of FANETs.

Moreover, FANET datasets for ETSC require a slightly different construction compared with other FANET datasets^24–26^. ETSC datasets require a label to be assigned to an ordered sequence of events, rather than a single data point. This differs from other FANET datasets, where labels are typically applied to averages or counts of metrics over fixed time periods^25,26^. For example, the dataset used in one study^25^ counts the number of forwarded route request packets^27^ in 5 second intervals. Conversely, one dataset assigned labels to individual packets or metrics instead of providing averaged values^24^. However, this method does not preserve event ordering, limiting its suitability for ETSC models.

Furthermore, we identified only one prior study that incorporated a GHA within a FANET dataset^26^. However, this study did not simulate varying types or intensities of GHAs, despite evidence from previous research showing that both factors can significantly affect the selection of defence strategies^4,5,28^. The *intensity* of a GHA refers to the packet dropping rate exercised by the attacker, which allows for more covert (lower intensity) or overt (higher intensity) attacks. Counter-intuitively, previous literature^4,5,28^ has shown that less intensive GHAs can harm the FANET more in the long term compared with more aggressive forms of GHA. This evidence suggests that accurately classifying a range of GHA types and intensities is vital for enabling a more targeted and effective defence strategy.

We integrated these three essential properties into our proposed dataset, FAN-GHETS24^29^, the **FAN**et **G**rey **H**ole dataset for **E**arly **T**ime **S**eries classification. These properties are realised through three key contributions that define the design of our dataset. Firstly, we use the NS-3 simulator^30^ as a robust and widely accepted testbed, offering a realistic and flexible environment for conducting FANET experiments. Secondly, we construct the dataset using sequences of packet-level interactions between pairs of nodes, where each sequence is labelled according to the behaviour of the node being interacted with. This structure captures temporal patterns essential for training ETSC models. Thirdly, we simulate a diverse range of grey hole attacks by introducing malicious nodes with varying attack types and intensities. This diversity ensures that the resulting models can generalize effectively across different adversarial behaviours. Table 1 summarises the datasets we have reviewed and indicates the attractive properties of FAN-GHETS24^29^. For clarity, dataset names which are in italics are “given names”, that is, a name which has been assigned to that dataset in the absence of any other name.

**Table 1:** Comparison of Datasets.

Dataset Name	Suitable for FANET Environments	Suitable for ETSC Models	Grey Hole Attack Type and Intensity Included	Publicly Available
KDDcup’99^18^	✗	✓	✗	✓
NSL-KDD^19^	✗	✓	✗	✓
CIC-IDS2017^20^	✗	*✓*	✗	*✓*
WSN-DS^68^	✗	✗	✗	*✓*
*FL-IDSDS*^25^	*✓*	✗	✗	✗
*ETD-DS*^24^	*✓*	✗	✗	✗
FAN-GHETS24^29^	*✓*	*✓*	*✓*	*✓*

We use NS-3^30^ to simulate the mobility and low level networking components of the FANET environment to generate FAN-GHETS24^29^. We choose NS-3 due to its extensive documentation, its realistic representation of ad hoc environments^31^ and its prevalence within the field of ad hoc security^32–34^. Unfortunately, NS-3 has a large learning curve and requires advanced C++ skills to create complex FANET environments. This restricts machine learning and computer networking specialists who may not have the time or resources to build a complex simulation to test their ETSC algorithms. Ergo, this dataset provides access to the FANET security research area without the need for proficiency in network simulation software.

In summary, we have highlighted the need for a specific FANET dataset for the training of early time series classification models to recognise different GHA types. The results from our validation section shows that the dataset is well suited for these models. However, the dataset also presents realistic and significant challenges for ETSC models. These challenges include: 1) the accurate and timely classification of long time series data, 2) the identification of subtle differences between classes amongst large amounts of potentially unrelated data and 3) a large and unbalanced dataset. This dataset enables future researchers to develop sophisticated and fast detection models that can defend against multiple types and intensities of GHAs.

## Methods

This section is split into two distinct subsections. Firstly, we discuss the FANET simulation needed to gather metrics for the creation of the dataset. Secondly, we describe how the simulation output and post processing methods form the finished dataset.

### Simulation

To increase the realism of our dataset, we present a motivating scenario in the form of an urban search and rescue (SAR) use case. Next, using this scenario, we define the set of simulation parameters based on UAV and FANET specifications. Once our environment has been setup, we then introduce the 11 variations of grey hole attack which are integrated into the simulation. Lastly, we summarise how the simulation is executed and how the information logging module enables us to capture metrics from the simulation.

#### Search and Rescue Scenario

Our hypothetical SAR scenario is based on a post-earthquake natural disaster. Such an earthquake destroys fixed communications infrastructure, buildings and, critically, endangers human life. In response to this scenario, search and rescue teams are deployed and have numerous tasks, such as locating survivors, evaluating building structure, and transporting supplies. Given the unfavourable environment conditions, these tasks come with considerable risk if manned SAR teams were dispatched. Therefore, UAVs are deployed in their stead. UAVs have been shown to be adept at many of these SAR tasks. For example, they have proven their ability to assess building stability using 3D point clouds^35^ or assess disaster scenes using fast 3D modelling approaches^36^. In addition, UAVs have also proven useful in the field of humanitarian logistics by transporting medical supplies to displaced people^37^. As the aforementioned SAR tasks require different sensors and equipment, multiple UAVs are dispatched simultaneously and form a FANET. Typically, the FANET is controlled by a ground crew that operates from a *Ground Control Station* (GCS)^38^. However, these UAVs are expected to be somewhat autonomous to allow the GCS crew to perform other tasks simultaneously^39^. The GCS is represented in our simulations as a stationary node called the *GCS node*.

Some SAR tasks can be carried out by the drones independently, but, other tasks, such as voice communication between the ground crew and victims^40^, will need the support of the entire FANET. To this end, our scenario stipulates that a UAV must remain with the victim in order to enable voice communication between the victim and the GCS. This UAV is named the *static node* within our simulation. This communication line is possible with the establishment of multi-hop routes via the ad-hoc on-demand distance vector (AODV)^41^ protocol, which uses the other UAVs in the network as routers. The AODV protocol is used for UAV-to-UAV communication as well as UAV-to-GCS and GCS-to-UAV communication. A fundamental requirement of these networks is to maintain the quality of service (QoS) so the survivor and the GCS crew can remain in contact while other SAR tasks are carried out.

#### UAV Specification

Due to the diverse SAR tasks described previously, UAVs of differing capabilities are required. There are a number of UAVs designed for these kinds of applications. We briefly describe three of them: the DJI Matrice 300 RTK^42^, the Inspired Flight IF1200^43^ and the Skyfront Perimeter 8^44^. The SkyFront Perimeter 8 boasts a large carrying capacity of 23 kg with a comparatively long battery life of 60 mins compared with other UAV models. This makes the Skyfront Perimeter 8 suitable for humanitarian logistics tasks. On the other hand, the Inspired Flight IF1200 has a fast horizontal speed of 25 m/s, making it suitable for quickly assessing building stability. In contrast, the DJI Matrice 300 RTK boasts a compact design, enabling it to navigate through tight spaces with ease.

The communication range of UAVs within simulated FANET environments is very broad, with some published work simulating communication ranges of 30m^45^ to 1000m^46^. Furthermore, depending on the category, the standards for 802.11 wireless communication enables a communication range between 80m and 1000m^47^. For search and rescue operations, it is desirable for communication distances to be as large as possible to ensure adequate UAV coverage of the area^48^. However, they are limited by, amongst other factors, line of sight (LoS). Studies with higher ranges do not take into consideration non-LoS communication distances, i.e. practical communication ranges of UAVs deployed in real life scenarios. The physical layout of the environment is inherently hard to predict and simulate, therefore, we provide a communication range which is both similar to previous works^28,32^ and also reflects projections based on studies involving extended-range, non-LoS communication scenarios^47^. In our simulation, we choose a maximum communication range of 380 m.

We have restricted our simulation time to fall in line with the minimum battery life of these UAVs, as we do not consider recharge time within these simulations. The Inspired Flight IF1200^43^ has a stated battery life of 24 minutes at maximum take off weight. Therefore, we use 20 minutes as a conservative simulation time.

Each UAV has the potential to enable *promiscuous mode*, which allows the UAV to receive all packets which are being sent by other UAVs in their communication range. These packets provide some valuable context, as nodes are able to “listen in" on communications between neighbouring nodes. In addition, all communication links between UAVs are assumed to be bi-directional.

In terms of maximum node speed, most studies use a range between 0 and 60 m/s^49–52^. However, considering that the UAVs reviewed previously have a maximum speed of 25 m/s, we revised this speed range to 0-35 m/s to include any other UAV models which may have a slightly higher maximum speed.

Every UAV in the simulation has the ability to measure their own speed and position, as well as the positions and speeds of neighbouring nodes. Similar to a previous work^53^, this information can be summarised in two forms: *distance* and *direction*. The distance, *d*_*ij*_(*t*_*k*_), is defined as the euclidean distance between any node, *i*, and another node, *j* at time *t*_*k*_. Moreover, we use relative distance measurements between time steps, to define direction:

1 $$\Delta {d}_{ij}({t}_{k})={d}_{ij}({t}_{k})-{d}_{ij}({t}_{k-1}),$$

which indicates if the two nodes are gradually moving away or moving towards each other. Direction and distance values are computed every 100ms.

#### Flying Ad-hoc Network (FANET) Specification

As mentioned previously, UAVs use the AODV protocol^41^ to route data packets. AODV is a popular routing protocol, known for its low network overhead and its high throughput in FANET environments^54^. Hence, we use the AODV protocol in this work.

**Fig. 1:**
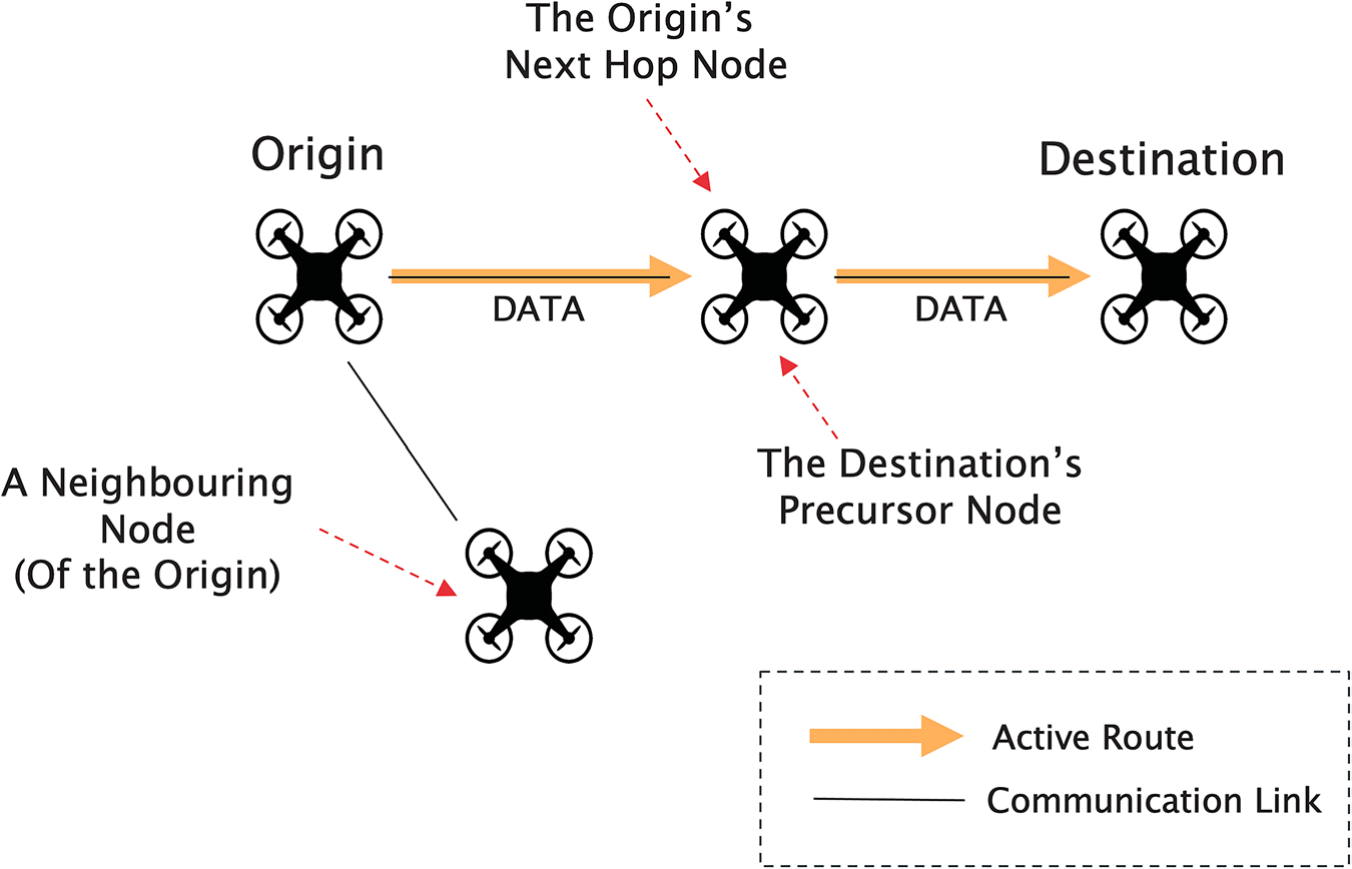
This Figure shows a simple 4 node FANET and the routing between the Origin and Destination node. Specific routing terms such as Next Hop, Precursor Node and Neighbouring Node are also indicated.

When discussing routing within an ad-hoc setting, it is helpful to define certain terms used to identify specific features of FANETs. Specifically, sending a packet to the next node in the route constitutes the *next hop* node. A *sender* and *receiver* imply a direct, one-hop connection where data is transmitted from the sender to the receiver without any intermediate nodes. In contrast, an *origin* or *destination* node refers to the endpoints of a multi-hop connection, where data packets travel through one or more intermediate nodes before reaching their final destination. The *origin* node is the initial sender of the packets, while the *destination* node is the final receiver. A *neighboring node* is any node that is within direct communication range of a given node and is capable of exchanging data without intermediate hops. Furthermore, a *precursor node* refers to the previous node along the route which also known as the *previous hop*. Understanding these terms is crucial for comprehending the dynamics of data transfer and route maintenance in FANETs. Figure 1 provides a visual summary of these routing concepts, illustrating the roles of next hop, sender, receiver, origin, destination and precursor nodes within a FANET.

We include AODV message types in our dataset because they can serve as valuable indicators of the network’s state. To highlight the importance of storing these message types, we provide a brief explanation of how the AODV protocol routes data in a FANET. AODV is a reactive protocol, meaning routes are established whenever data transfer is needed. AODV establishes routes via the communication of three main types of control message: RREP, RREQ and RERR. In more detail, RREQ or Route Request messages are broadcast packets which are first generated by the origin node who wishes to find a particular destination node. While broadcasting, the nodes setup a reverse route in the routing tables to keep a record of the route if the destination node is found. Seeing that a route is possible between the origin and destination, an RREP or Route Reply message is unicast along the reverse route. Finally, the origin node assesses all of the received RREPs and a route with the lowest hop count is chosen. With the decision made, the route is now marked as *valid* and can be used to forward data packets. To ensure that neighbouring links are maintained, *HELLO* messages are broadcast at a fixed interval. Due to the mobility of UAVs within FANETs, link breaks are common, and therefore, an RERR or route error message is generated and sent to all *precursor nodes* to inform precursor nodes that the route is now invalid and should not be used. HELLO messages are also used to exchange UAV position information.

In addition to routing, we must also decide how the UAVs within the FANET will move. We have chosen to implement the random way point (RWP) mobility model for the mobile nodes within our simulation due to its suitability for search and rescue scenarios^3,54^. From our application scenario, UAVs execute their SAR tasks autonomously, which results in mobility patterns that resemble the RWP mobility model.

The UAV density in FANETs is lower than other ad-hoc network paradigms such as vehicular ad-hoc networks (VANETs) or mobile ad-hoc networks (MANETs)^54^. Therefore, we choose to simulate 24 mobile UAVs, as most studies place the number of UAVs in a FANET between 5 and 35^46,49,50^. The FANET is simulated with three different sets of *attack node ratios* (ANR), which we call *environments*. ANR is the ratio of attacking nodes to the number of benign nodes within the simulation. The three environments are summarised in Table 2.

**Table 2:** Environments Considered in this Work.

Num. Attack Nodes	Num. Benign Nodes	Attack Node Ratio
3	21	12.5%
6	18	25%
9	16	37.5%

#### Grey Hole Threat

We assume that a proportion of these UAVs have had a grey hole attack introduced in their networking software. When simulation commences, one attack type and intensity value is selected for all attacking nodes within the simulated FANET. The type and intensity remains fixed for the entire length of the simulation. Furthermore, we do not consider collusion between attacking nodes. Three types of grey hole attack are simulated based on previous literature:^55,56^

1. Firstly, the ***probabilistic*** grey hole attack drops packets probabilistically with probability *p* ∈ [0, 1]. This is enforced in every instance where a data packet is being forwarded by the grey hole node.
2. Secondly, The ***time***-***based*** grey hole attack drops all data packets within a specific time interval. For this attack, the simulation is divided up into time windows of length *w*. The variable, *b* ∈ [0, 1], determines the probability that a time window will be a *drop window*. Within the drop window, if a request to forward a data packet is made, the data packet is dropped.
3. Thirdly, the ***precursor***-***based*** grey hole attack drops all data packets from a specific precursor node in the routing path. To explain further, every node in the network at the start of the simulation is assigned a *drop-node* status with probability *q* ∈ [0, 1]. The drop node status, which persists for the entire simulation, is a boolean variable that indicates that a specific node will have its data packets dropped by the grey hole node if it acts as a precursor. In addition, each attacker node will have a unique assignment of drop-node statuses and, as we assume there is no collusion between attacking nodes, the drop node status can be assigned to attacking nodes as the attacking nodes do not know the identities of other attacking nodes.

In addition, please see Algorithms 1, 2 and 3 for a more specific description of the attack dynamics of these grey hole attacks.

##### Algorithm 1

Probabilistic Grey Hole Attack.

##### Algorithm 2

Time-based Grey Hole Attack.

##### Algorithm 3

Precursor-based Grey Hole Attack.

We have also implemented three intensity values for the Precursor-Based and Time-Based attack while the Probabilistic grey hole attack has five intensity values. A range of Probabilistic grey hole attacks are common in current literature^5,28^, hence the number of probabilistic attacks in our work are proportionally larger. In addition, we found through experimentation that these intensity intervals were sufficient enough to be useful whilst also avoiding the redundancy of having overly small intervals, ensuring that each interval represents a meaningful distinction for classification purposes. To keep explanations concise, we will refer to the unique combination of grey hole attack and intensity by their short form name. These short form names are included in the set of class labels, in addition to the benign class label which represents a benign node. Table 3 summarises the attacks used in this study, detailing their attack type, short form name and any associated variable(s).

**Table 3:** Class Labels Present in the Dataset.

Grey Hole Attack Type	Short Form	Variables
None	**Benign**	—
Probabilistic	**PD-0.2**	*p* = 0.2
Probabilistic	**PD-0.4**	*p* = 0.4
Probabilistic	**PD-0.6**	*p* = 0.6
Probabilistic	**PD-0.8**	*p* = 0.8
Probabilistic	**PD-1.0**	*p* = 1.0
Time-based	**Tdrp-0.3**	*b* = 0.3 *w* = 5 seconds
Time-based	**Tdrp-0.5**	*b* = 0.5 *w* = 5 seconds
Time-based	**Tdrp-0.7**	*b* = 0.7 *w* = 5 seconds
Precursor-based	**Sele-0.3**	*q* = 0.3
Precursor-based	**Sele-0.5**	*q* = 0.5
Precursor-based	**Sele-0.7**	*q* = 0.7

#### Simulation Area and Attack Integration

All simulations contain the GCS and static node which are placed at either end of a 1050 m × 150 m simulation area. The simulation area is split vertically into three sub-areas of equal sizes where each sub-area divides the number of attacking and benign nodes equally. For this reason, the number of UAVs in our simulation is a multiple of 3. We also wanted periods of time within the simulation where the origin and destination node would not necessarily have a path (as would be the case in real world scenarios). Refining the UAV number further, we decided against the upper range of 33 UAVs and decided to implement 24 to encourage some sparsity. In addition, all mobile nodes are strictly bound to a specific sub-area, however, they are allowed to move freely within their designated area. This setup, similar to previous works^57,58^, ensures that data originating from the GCS traverses to the other side of the network via multi-hop routes consisting of two or more forwarding nodes, which encourages multi-hop communication routes.

**Fig. 2:**
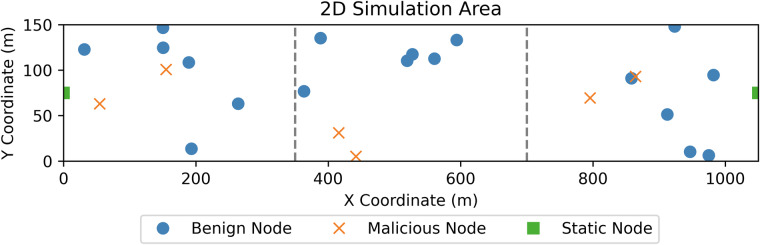
This figure shows the simulation area which also shows an example distribution of benign and malicious. Static and GCS nodes are shown at either ends of the simulation area.

At the start of the simulation, the malicious nodes are all assigned one specific grey hole attack class whereas benign nodes can only be assigned the benign class. Each grey hole attack from Table 3 is simulated 10 times within three environments, making 330 simulations in total. Figure 2 provides an example simulation environment and Table 4 summarises the simulation parameters.

**Table 4:** NS-3 Simulation Parameters.

Parameter	Value
Simulation Time	1200 Seconds
Static UAV	1
GCS Station	1
Simulation Area	1050 m × 150 m
Mobility Model	Random Way Point
UAV Communication Range	380 m

#### Information Logging

Packet information is collected at the network layer through two routes: 1) through the forwarding function of the protocol and 2) via promiscuous mode. Only benign nodes collect traffic from the network layer, as malicious nodes do not classify other nodes in the network and therefore have no incentive to collect such data. If a packet is received via promiscuous mode, we say it is *witnessed* by a particular node and is recorded in the log file. To clarify, we define a *record* as a single packet sent, received, or witnessed that contains 13 metrics related to that particular packet. These 13 metrics are summarised in Table 5.

**Table 5:** Extracted Data From Simulations.

Extracted Data	Feature Engineering Technique	Brief Description
Timestamp	One-step difference & Normalisation	This represents the current simulation time of when the packet was sent, received or witnessed. In post processing, this represents the time elapsed since the last packet was sent, received or witnessed.
**Seen By** IP address	Anonymization & Dummy Encoding	This is the IP address of the node which sent, received or witnessed this packet. Packets are witnessed by using promiscuous mode.
**Sent To** IP address	Anonymization & Dummy Encoding	This is the IP address of the node which the packet is being sent to. If routing table information is available, then this takes the value of the next hop IP address.
**Sent By** IP Address	Anonymization & Dummy Encoding	This is the IP address of the node which sent this packet.
Network Packet Type	Dummy Encoding	This is a enumerated type which indicates if the packet is an RREP, RREQ, RERR or data packet
Packet ID	Normalisation	This is the value of the packet identification field in the IP header of the packet.
TTL	Normalisation	This is the time-to-live value of the packet.
Is Promiscuous Mode	None	This indicates if the packet was received via promiscuous mode.
Node’s Current Speed	Normalisation	This is the current speed of the node in m/s.
Distance to **Sent To** IP Address	Normalisation	This is the euclidean distance from the current node to the node indicated by **Sent To**. **Sent To** can also be broadcast; in this case, the value is 0.
Direction to **Sent To** IP Address	Normalisation	This is the direction (as defined in Equation (1)) from the current node to the node indicated by **Sent To**. **Sent To** can also be broadcast; in this case, the value is 0.
Distance to **Sent By** IP Address	Normalisation	This is the euclidean distance from the current node to the node indicated by **Sent By**. **Sent By** can also be the node’s own IP address; in this case, the value is 0.
Direction to **Sent By** IP Address	Normalisation	This is the direction (as defined in Equation (1)) from the current node to the node indicated by **Sent By**. **Sent By** can also be the node’s own IP address; in this case, the value is 0.

We have created a custom AODV module which allows the logging of these packet metrics. Moreover, due to the availability of routing tables and mobility models, the packet metrics are fused with the information available from these sources. This contrasts with other traditional approaches, such as PCAP tracing, which typically does not store this information. Specifically, when PCAP traces show a data packet routed via AODV, the IP address of the next hop node is not stored within the header, as all information regarding the route is stored in the routing tables of the individual nodes. Both the sender and receiver node have information regarding which node they need to forward to or receive from, which means that this information does not need to be stored in the header of the data packet. By using logging statements within the AODV routing module, we can extract these particular IP addresses by accessing the routing table. And, as mentioned previously, PCAP traces do not store mobility information, therefore, it is difficult to determine from a PCAP file if packet dropping is due to topology changes or malicious behaviour.

The records within our simulation are timestamped, enabling the preservation of packet ordering. The AODV control messages are extracted to establish situational awareness regarding routes and broken connections.

The IP addresses associated with all control and data packets are used to identify which nodes are sending and receiving data. These node IP addresses are stored as three main metrics:

- The **Seen By** IP address is the node which has sent, received or witnessed a packet.
- The **Sent By** IP address is the node which has sent this packet according to the “Seen by” IP address.
- The **Sent To** IP address is the node which the **Sent By** IP address has sent its packet to (from the point of view of the **Seen By** IP). The **Sent By** IP address can also be the broadcast address.

Mobility is an important metric for the identification of grey hole attack in FANET environments. Therefore, the directions and distances from the **Seen By** node to the **Sent To** and from the **Seen By** node to the **Sent By** node are recorded at the time the packet is sent, received or witnessed by the **Seen By** node. Of course, some of these packets are sent to a broadcast address and, as a node is not associated with this address, the direction and distance are set to 0m. Similarly, if the **Seen By** node is the same as the **Sent By** node, then the distance and direction are set to 0. There are also other situations where distance and direction need to be set manually. For example, if an IP address is out of range, then the distance is limited to the maximum communication range of the UAVs and the direction is set to 0m.

The metrics discussed thus far are essential for contextual awareness and node identification. To enhance grey hole threat classification, we introduce two additional metrics: time-to-live (TTL) and Packet ID. These features are commonly used in trust-based defence protocols^59^ to identify malicious behavior, and they serve a similar purpose here. The packetID and TTL allow cross-referencing occurrences of the same packet within the sequence, enabling the identification of repeated interactions and packet lifetimes as they move through the network. While the specific values of packetID and TTL are not significant on their own, tracking where identical packetIDs reappear and observing TTL decrements across the sequence provide crucial insights into node interactions and potential threat patterns.

We have also created a custom attack AODV protocol which drops data packets according to the grey hole attack specified at the start of the simulation. In addition, the attack AODV protocol has its own logging procedure which records the timestamp of every packet dropped. This provides useful information when analysing the final results of an ETSC IDS model.

### Dataset Creation

#### Node Perspective and Anonymization

Our dataset consists of sequences of interactions between any benign node in the network, which we will refer to as the *eval node*, and any other node within the communication range of the eval node which is the subject of the evaluation, referred to as the *subject node*. The eval node investigates the subject node by collecting their packet interactions and any promiscuous information related to the subject node. Through these interactions, the eval node can gradually accumulate evidence to classify the subject node into a particular category.

So far, we have described the data collection process that captures all node communications across the entire FANET. This raw data must be further processed to construct sequences that represent the complete history of interactions between specific eval-subject node pairs. To illustrate this process, we focus on a single eval node, which generates one or more sequences depending on the subject nodes it interacts with. The same procedure is repeated for each eval node in the FANET. It is important to emphasize that eval nodes are always benign, while subject nodes may be either benign or malicious. Accordingly, each sequence is labelled based on the class of the subject node. The processing steps are outlined below:

1. **Filter by Evaluation Node Perspective**: Firstly, the data is filtered so that the IP addresses in the **Seen By** column is the IP address of the eval node. The resultant data contains packet interactions which have been sent, received or witnessed by the eval node. The output of this process is known as the eval data. As each record in the Seen By field now contains the same value, this can be safely removed.
2. **Group by Contacted Subject Nodes**: The eval data is further segmented based on the set of nodes that the eval node has encountered during the simulation. Each of these contacted nodes becomes a distinct subject node. So, for each eval-subject node pair, we apply the following procedure:
  - **Sent To Filtering**: The eval data is filtered according to the Sent To field, where the Sent To IP address is equal to the IP address of the subject node, origin node, destination node or the broadcast address.
  - **Sent By Filtering**: Separately, the eval data is also filtered according to the Sent By field, where the Sent By IP address is equal to the IP address of the subject node, origin node or destination node.
  - **Concatenation**: The Sent By and Sent To filter data is then concatenated. We refer to to this output as the concatenated data.
  - **Anonymisation**: We apply an anonymisation procedure which replaces IP addresses with string variables. Anonymizing the sequences means that they are IP address agnostic, therefore, any trained model can be integrated into any node within the FANET. Every IP address in the Sent To and Sent By fields of the concatenated data is substituted with “MY_IP”, “INSPECT_IP”, “STATIC_NODE_IP”, “GROUND_CONTROL_IP”, “BROADCAST_IP” or “OTHER_IP”. “OTHER_IP” can be thought of as a catch-all string representation which substitutes any IP address if the IP address has not already been substituted by a previous string value. See Table 6 for an example of how IP addresses are substituted with string values.

**Table 6:** This table describes the string values which are substituted for specific IP addresses during the anonymisation procedure.

IP Address (Example)	String Replacement	Description
10.1.1.8	MY_IP	An Eval Node IP
10.1.1.14	INSPECT_IP	A Subject Node IP (Benign)
10.1.1.26	STATIC_NODE_IP	The Static Node IP
10.1.1.25	GROUND_CONTROL_IP	The GCS Node IP
10.1.1.255	BROADCAST_IP	The Broadcast Address
10.1.1.9	OTHER_IP	A Benign Node’s IP Address
10.1.1.22	OTHER_IP	A Malicious Node’s IP Address

We give example IP addresses here to illustrate how these addresses are substituted. For example, in this instance, the subject node is a benign node and the other IP addresses (one benign and one malicious) have been substituted as “Other”.
  - **Label Sequence by Subject Node Class**: This generated sequence is then labelled according to the class of the corresponding subject node.

While effective, this node perspective and anonymisation approach does introduce some limitations. For example, 3rd party information, i.e. information from other sequences, cannot be used to classify a particular subject node due to the anonymisation process. Moreover, the lack of routing context also means that packets cannot be tracked through the simulation. More practically, in real world deployments, a log would have to be continuously filtered, anonymised and stored by the UAV in order to feed the IDS system. If an eval UAV has many neighbouring UAVs, the filtering process could consume significant resources.

#### Feature Engineering

It has been shown that utilising a machine learning based approach for early classification is effective when the training dataset is large^60^. Hence, we expect the majority of researchers working with our dataset to use machine learning models. To aid these researchers, we enhance the suitability of our dataset for machine learning models by applying feature engineering techniques^61^. The metrics from our simulation encompass various data types, each necessitating specific processing methods. Please refer to Table 5 for a description of the data processing methods used for each data field. In the remaining paragraphs of this section, we give more detail of why these feature engineering techniques are used. Many of these metrics, such as direction, distance, TTL and speed are simply normalised. In contrast, other feature engineering techniques require more depth explanation.

We find that the time difference between timestamps can provide contextual meaning, as the inter-packet time delay can infer packet loss patterns over time or the freshness of routing paths. So, we include the time differences between packets as a feature within our dataset. We then normalise these timestamp differences to produce the final feature.

Due to the anonymization process, each IP address field now has a fixed number of potential values, therefore, we can implement dummy encoding^61^ for the **Sent To** and **Sent By**. Notice how the **Seen By** node is not included in the process, as once the anonymization process is completed, the IP address of the **Seen By** node does not add any information. Furthermore, we can also implement dummy encoding for the network packet type metric as that metric specifies if the packet is an AODV control message or a data packet. Overall, the implementation of dummy encoding means that the unique values in these fields are now represented by a sparse matrix of binary values. This is beneficial for machine learning algorithms, but also increases the dimensionality of the feature space.

## Data Records

The dataset is available in compressed form via this link: 10.5281/zenodo.13315419, with this section being the primary source of information on the availability and content of the data being described.

**Fig. 3:**
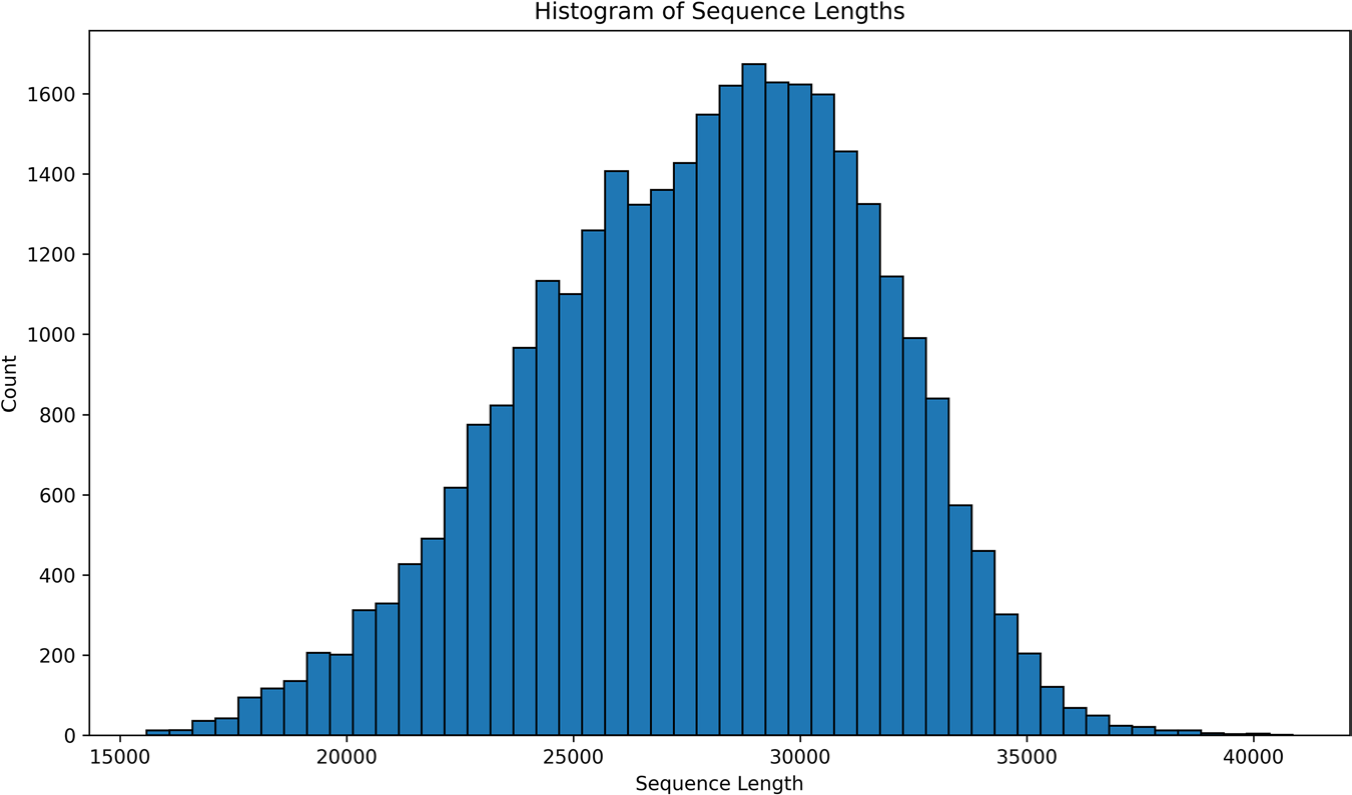
This graph shows a histogram of sequence lengths in FAN-GHETS24^29^.

**Fig. 4:**
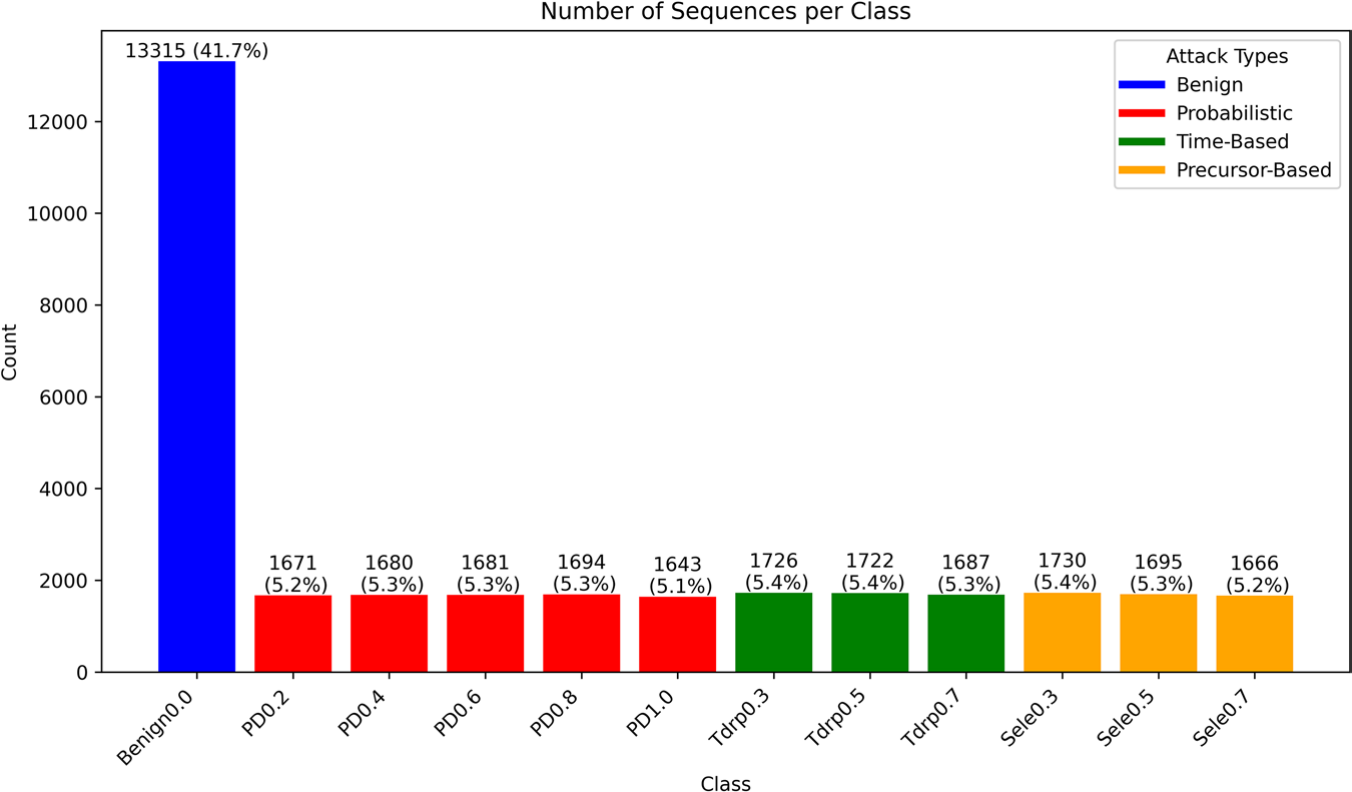
The bar chart illustrates the distribution of sequences across different classes in FAN-GHETS24^29^, with each color representing a distinct attack type. Blue denotes Benign traffic, red represents Probabilistic (PD) attacks, green corresponds to Time-based (Tdrp) attacks, and orange indicates Precursor-based (Sele) attacks. The height of each bar reflects the total number of sequences for the respective class. The values displayed above each bar indicate both the absolute count of sequences and their percentage relative to the total dataset.

The FAN-GHETS24^29^ dataset consists of 31910 sequences across 12 classes. The distribution of classes is shown in Fig. 4, which predictably shows that the benign class is in the majority, as one would expect in cyber security scenarios. In addition, the distribution of sequence lengths in Fig. 3 shows that the majority of sequences in FAN-GHETS24^29^ have longer lengths than what is used in the validation model. This means that there is potential to build larger models capable of handling longer sequences, which could improve the performance of future ETSC algorithms.

With the feature engineering techniques implemented, a single data point or record in any sequence consists of 8 continuous features, represented by the float data type, and 17 categorical features, represented by the boolean data type. In total, there are 25 features. Table 7 shows 4 example raw outputs from the simulation which can be processed into records via the techniques described previously. Table 8 shows 4 example records from the dataset which have not been generated from the raw output in Table 7. For every sequence in the dataset, there are two labels: 1) the grey hole attack type and 2) the grey hole attack intensity. The labelling has been divided to enable different types of classification models to be trained. For example, one may wish to train a model to recognize only the type of grey hole attack, rather than both the type and intensity of the attack.

**Table 7:** This table shows 4 records from the simulation output.

Metric	Raw Output 1	Raw Output 2	Raw Output 3	Raw Output 4
**timestamp**	1013304127	1013304165	1013304253	1013304263
**seen_by**	10.1.1.3	10.1.1.5	10.1.1.6	10.1.1.4
**sent_to**	10.1.1.255	10.1.1.255	10.1.1.255	10.1.1.255
**sent_by**	10.1.1.1	10.1.1.1	10.1.1.1	10.1.1.1
**net_packet_type**	2	2	2	2
**packet_id**	− 1	− 1	− 1	− 1
**ttl**	1	1	1	1
**promisc_mode**	1	1	1	1
**current_speed**	0.040265	0.496471	0.576345	0.24888
**sent_to_d_distance**	0	0	0	0
**sent_to_distance**	0	0	0	0
**sent_by_d_distance**	0.0203302	− 0.217512	− 0.222136	− 0.0804193
**sent_by_distance**	0.0357778	0.0472725	0.0717229	0.0748423

These four packets are the result of a single broadcast message from 10.1.1.1, which was witnessed by four different nodes. The broadcast message is an AODV RREP message with a TTL of 1, indicating that this is a HELLO message.

**Table 8:** This table shows 4 example data records from one sequence.

Feature	Type	Record 1 (*X*_*t*,:_)	Record 2 (*X*_(*t*+1),:_)	Record 3 (*X*_(*t*+2),:_)	Record 4 (*X*_(*t*+3),:_)
net_packet_type_DATA_PACKET	Categorical	1.00000	1.00000	1.00000	1.00000
net_packet_type_RERR_PACKET	Categorical	0.00000	0.00000	0.00000	0.00000
net_packet_type_RREP_ACK_PACKET	Categorical	0.00000	0.00000	0.00000	0.00000
net_packet_type_RREP_PACKET	Categorical	0.00000	0.00000	0.00000	0.00000
net_packet_type_RREQ_PACKET	Categorical	0.00000	0.00000	0.00000	0.00000
promisc_mode	Categorical	1.00000	1.00000	1.00000	1.00000
sent_by_GCS_IP	Categorical	0.00000	0.00000	0.00000	0.00000
sent_by_INSPECT_IP	Categorical	0.00000	0.00000	0.00000	0.00000
sent_by_MY_IP	Categorical	0.00000	0.00000	0.00000	0.00000
sent_by_OTHER_IP	Categorical	0.00000	0.00000	0.00000	0.00000
sent_by_STATIC_NODE_IP	Categorical	1.00000	1.00000	1.00000	1.00000
sent_to_BROADCAST_IP	Categorical	0.00000	0.00000	0.00000	0.00000
sent_to_GCS_IP	Categorical	1.00000	1.00000	1.00000	1.00000
sent_to_INSPECT_IP	Categorical	0.00000	0.00000	0.00000	0.00000
sent_to_MY_IP	Categorical	0.00000	0.00000	0.00000	0.00000
sent_to_OTHER_IP	Categorical	0.00000	0.00000	0.00000	0.00000
sent_to_STATIC_NODE_IP	Categorical	0.00000	0.00000	0.00000	0.00000
current_speed	Continuous	0.37413	0.37413	0.37413	0.37413
packet_id	Continuous	0.27491	0.27501	0.27512	0.27522
sent_by_d_distance	Continuous	0.40920	0.40920	0.40920	0.40920
sent_by_distance	Continuous	0.62402	0.62402	0.62402	0.62402
sent_to_d_distance	Continuous	0.50000	0.50000	0.50000	0.50000
sent_to_distance	Continuous	1.00000	1.00000	1.00000	1.00000
timestamp difference	Continuous	0.00127	0.00330	0.00233	0.00181
ttl	Continuous	0.96923	0.96923	0.96923	0.96923

This specific sequence shows 4 data packets which have been sent from the static node to the GCS node. These packets have been witnessed via promiscuous mode, and, have been sent in rapid succession, as indicated the “timestamp difference” feature. This data is not the result of processing the simulation data from Table 7. Column notation will be described in a subsequent section.

The FAN-GHETS24^29^ dataset consists of sequentially numbered parquet files in the format: “example_host_data_*srID*.parquet", where *srID* is the sequence reference ID. Parquet files are a compressed format which are easily readable by the Pandas python library. Every parquet file is paired with a JSON file with the same sequence reference ID and has the file name format: “example_host_data_*srID*.json". This JSON file contains metadata regarding the parquet file of the same sequence ID. The metadata includes information regarding the total sequence length, the class which the subject node belongs to, a list of attack points (If applicable) and the feature data types. Attack points are specific time step values that indicate when a packet was dropped due to the presence of a GHA node.

For anyone wishing to recreate our dataset, we provide an explanation of the configuration files used for the simulation. The benign and attack configuration files are in the format “*.ini” and provide a list of benign or attack protocol parameters called *strategies*. The system will automatically run all unique combinations of attack and benign strategies. As is evident, we only have one benign strategy within the defence configuration file, which monitors and logs network information. However, if another monitoring protocol was developed, then this protocol can be specified in the benign configuration file. The same technique could also be used for the attack configuration file, if one wished to write a custom NS-3 AODV protocol which allows another form of attacking behaviour. We use a python script to merge the benign and attack configuration *.ini files and generate a simulation configuration file for every simulation which needs to be run.

Simulation configuration files specify a random seed and the environment parameters such as the maximum node speed and the number of attack nodes within the simulation. The random seed determines the placement, speed and direction of all mobile nodes as the random way point mobility model generates its mobility parameters from this random seed. We supply a template simulation configuration file, “SimulationConfig.ini” which can be modified if needed, however, we provide a python script named “RunSimulations.py” which generates a set of simulation configuration files and automatically runs with with the compiled NS-3 executable. These simulations run in parallel, with each simulation producing two CSV files. The first CSV contains the raw data used for the creation of the dataset, and the second is an attack CSV file, which stores the timestamp of when an attack is executed.

The environment options within this file are limited to the SAR scenario. However, for users wishing to create a custom FANET scenario, we highlight three key functions in the “sim.cc” file where modifications can be made to support this.

Firstly, the Create_Nodes() function creates the nodes, malicious and benign node groups, and, assigns the nodes to particular areas of the simulation. This is also where users can customize the mobility model, initial node positions, movement speed, pause time, and the type of random distribution used for positioning. Secondly, the GenerateTraffic() function lets you customize the packet sending frequency, quantity, and source/destination sockets. One could modify this function to define a list of destination and origin sockets, allowing the simulation to send to and receive from multiple nodes. Finally, we would like to highlight the CommandSetup() function, where one can externalise new environment parameters from the previous two functions, enabling simulation changes without the need for recompilation.

While FAN-GHETS24^29^ provides the necessary sequence data for training ETSC models, it also presents some minor limitations. For instance, the high dimensionality of each record can restrict the number of applicable machine learning techniques which can use this data. For example, Random Forest or SVM classifiers are designed to use a much smaller number of features and are therefore inappropriate. In contrast, deep learning methods appear to be a robust choice of model, as demonstrated in the next section. However, we did find that Long Short-term Memory (LSTM) models struggled with the temporal dimension of FAN-GHETS24^29^. Another limitation of FAN-GHETS24^29^ is the simulation environment. Ideally, the simulation would incorporate more detailed aspects of wireless communication, such as multipath propagation caused by signal reflections from physical objects. However, modelling such phenomena would require a significantly more advanced simulation environment, involving elements like 3D trajectory planning and collision detection for the UAVs. This level of physical modelling falls beyond the scope of our work, as our work focuses on evaluating grey hole attacks from a networking and protocol-level perspective.

For clarification, we would like to point out that FAN-GHETS24^29^ and its source code repository does not contain any malicious code which could endanger a user’s computer or UAV network. The attack source code in the repository is specific to NS3 simulations and would have to be modified extensively for it to be a risk to real-world UAVs. Furthermore, the attacks presented in this article have been known for some time and therefore could not be used as part of a zero-day attack. Additionally, the data within FAN-GHETS24^29^ can be summarised as a log of interactions between two UAV entities, making it unsuitable for use as part of an attack.

## Technical Validation

Henceforth, sequences of node interactions are referred to as *multi-feature time series* (MTSs), representing interactions between the eval node and the subject node. Each MTS is classified based on the subject node’s behavior, which can be one of the 12 specific class labels from Table 3. The model produced as a result of this technical validation operates in three stages:

In Stage 1, an autoencoder is trained on individual time steps from the training dataset’s time series. The validation dataset is used to evaluate the performance of this autoencoder. And, the autoencoder serves as a preliminary step for all subsequent base classifiers.

In Stage 2, several convolutional neural network (CNN) models, or base classifiers, are trained to classify sequences of these compact feature representations. Each successive base classifier is responsible for classifying an increasing segment of the time series. For example, the first classifier might operate on the first *τ* = 2 time steps of an MTS, the next on *τ* = 4 time steps, the next on *τ* = 6, and so on. In this way, each classifier processes progressively larger sections of the time series, building upon the earlier segments to improve classification accuracy.

In Stage 3, the confidence of a classification is determined from both the classification result of an unseen MTS and the *confidence matrix* of the base classifier. The confidence matrix, generated using the training dataset, provides a measure of how reliable a classification is. To determine an appropriate classification threshold, a range of confidence threshold candidates is generated from the validation dataset. These thresholds indicate how confident a model must be to make a classification decision. If the confidence value exceeds the selected threshold, the time series is classified. Otherwise, the decision is deferred until more data is available. The optimal threshold is chosen by balancing classification accuracy with timeliness.

This model establishes a suitable benchmark for both Accuracy and Earliness, which can be used to evaluate future ETSC models built on this dataset. The remainder of this section provides a detailed description of the model and presents the results. Before delving into the model details, however, we first introduce some formal notation.

### ETSC Preliminaries

Each MTS consists of a number of time steps, *t*, where, at each *t*, a vector of features is present. The maximum defined sequence length, *T*, denotes the longest period over which an MTS can be classified, though earlier classifications are possible. To formalize this concept, we define an MTS, *X*, as follows:

**Definition** (Multi-feature Time Series (MTS)). An MTS, *X*, is defined as a matrix $X\in {{\mathbb{R}}}^{\tau \times | V| }$, where *V* is the set of features and *τ* is the length of the multi-feature time series where *τ*  ≤  *T*. Standard matrix indexing applies, i.e., *X*_*t*,:_ represents the vector of features at time step *t*, and *X*_:,*v*_ represents the time series of feature *v*, ∀*t* ∈ {1, 2, …, *τ*}.

Within the FAN-GHETS24^29^ dataset, MTS lengths may vary: sequences shorter than *T* are excluded from this analysis while those exceeding *T* are truncated to length *T*. In the definition of an MTS, *τ* plays two roles: it represents both the MTS length and the specific *classification point* at which an MTS can be classified. We define these points formally below:

**Definition** (Classification Points). A set of classification points, ${\mathcal{T}}\subseteq \{1,2,\ldots ,T\}$, represent the specific time steps where the MTS could be classified.

In real-time scenarios, this early classification model would wait for more data to arrive before the next classification is made. However, in FAN-GHETS24^29^, the complete MTS sequence, of length *T*, is available straight away. Therefore, we emulate the data collection process by masking the time steps from *τ* to *T*. Then, if the early classification algorithm requires another classification at the next *τ*, the next portion of time steps is made available to the model. Like other classification models, this validation model requires the FAN-GHETS24^29^ dataset is divided into three distinct subsets: Training (80%), Validation (10%), and Testing (10%), to provide evidence of model performance. These datasets are formally described below:

**Definition** (Dataset). Given a set of class labels, ${\mathcal{Y}}$, a collection of MTSs, **X** = ⟨*X*^(1)^, *X*^(2)^, …, *X*^(*n*)^⟩, and their associated class labels, **Y** = ⟨*y*^(1)^, *y*^(2)^, …, *y*^(*n*)^⟩, a dataset is defined as ${\bf{D}}=\langle \langle {X}^{(i)},{y}^{(i)}\rangle | {y}^{(i)}\in {\mathcal{Y}},i=1,2,\ldots ,n\rangle$, where *n* is the number of MTSs in the dataset. In this work, there are three datasets: **D**_train_, **D**_test_, **D**_validation_, which represent the training, testing, and validation datasets, respectively.

With these datasets defined, we proceed to train a set of base classifiers, each responsible for making a classification decision at every classification point. Specifically, classifying an MTS at a given point, *τ*, requires a function that maps the observed sequence up to *τ* to a class label. This function is formalized below:

**Definition** (Classification of an MTS). Classifying an MTS at classification point *τ* involves finding a function ${{\mathcal{H}}}_{\tau }:{{\mathbb{R}}}^{\tau \times | V| }\to {\mathcal{Y}}$ that maps the first *τ* time steps of the MTS to its corresponding class label. An unscripted ${\mathcal{H}}$ defines the set of all base classifiers across all classification points, i.e., ${\mathcal{H}}=\{{{\mathcal{H}}}_{\tau }| \forall \,\tau \in {\mathcal{T}}\,\}$.

To simplify the notation, when classifying an MTS sequence with the classification function, ${\mathcal{H}}()$, the size of the MTS required is given by the subscript of the classifier. Hence, ${{\mathcal{H}}}_{\tau }({X}_{0:\tau ,:})$ and ${{\mathcal{H}}}_{\tau }(X)$ are equivalent. We define the early classification of an MTS using a function that identifies both the class label and the specific classification point at which the decision was made:

**Definition** (Early Classification of an MTS). Given a set of base classifiers, ${\mathcal{H}}$, the early classification of an MTS involves finding a function ${\mathcal{F}}:{{\mathbb{R}}}^{T\times | V| }\to \langle {\mathcal{Y}},{\mathcal{T}}\,\rangle$. We define ${{\mathcal{F}}}^{{\mathcal{Y}}}$ as the component of ${\mathcal{F}}$ that represents the classification output, ${\mathcal{Y}}$, and ${{\mathcal{F}}}^{{\mathcal{T}}}$ as the component that represents the classification point, ${\mathcal{T}}$, at which the classification is made.

The early classification model is evaluated with the test dataset using two criteria: Accuracy and Earliness, as described below:

**Definition** (Accuracy). Accuracy is the proportion of correctly classified MTSs compared to the total number of MTSs within a dataset, **D**, and is given by:

2 $$Accuracy({\mathcal{F}}\;)=\frac{| | \left\{\{i\in \{1,2,\ldots ,n\}\,| \,{{\mathcal{F}}}^{{\mathcal{Y}}}({X}^{(i)})={y}^{(i)}\}\right\}| | }{n},$$

where ⟨*X*^(*i*)^, *y*^(*i*)^⟩ ∈ **D**_test_ and *n* represents the number of MTSs in **D**_test_.

**Definition** (Earliness). Earliness is a measure of how early the ETSC model classifies a result. Earliness is given by:

3 $$Earliness({\mathcal{F}}\;)=\frac{1}{n}\mathop{\sum }\limits_{i=1}^{n}\frac{{{\mathcal{F}}}^{{\mathcal{T}}}({X}^{(i)})}{T},$$

where *X*^(*i*)^ ∈ **D**_test_ and *n* represents the number of MTSs in **D**_test_.

### Dimensionality Reduction

Dimensionality reduction ensures that the base classifiers operate on a low-dimensional representation of the data, making training more efficient. The autoencoder^62^ architecture is used here to perform this dimensionality reduction. Specifically, an autoencoder is comprised of two parts, an *encoder*, ${\mathcal{E}}$(.), and a *decoder*, ${\mathcal{D}}$(.), where the output of the encoder, *h*, is a latent variable which represents the encoded input, *s*: $h={\mathcal{E}}(s)$. To ensure that *h* represents a reduced form of *s*, a decoder function, $\widehat{s}={\mathcal{D}}(h)$, uses the latent representation as input and attempts to recreate the original input. Training of the autoencoder is then a case of minimising the reconstruction loss, ${L}_{{\rm{recon}}}(s,\widehat{s})$, using standard backpropogation and batch training techniques.

The input features to the autoencoder contain two different types of data, categorical and continuous, therefore, we found it pertinent to introduce a loss function for each type. Specifically, we introduce a categorical loss function, *L*_cat_, and a continuous loss function, *L*_con_, making the reconstruction loss the total of both losses:

4 $${L}_{{\rm{recon}}}={L}_{{\rm{cat}}}+{L}_{{\rm{con}}},$$

where *L*_con_ is the mean squared error loss function and the *L*_cat_ is the BCELossLogits loss function.

The autoencoder architecture used in this work is described in Table 9 and comprises a multi-branch encoder design which allows different feature types to be processed through the network separately, before being concatenated and reduced to the embedding dimension. Likewise, the decoder has a multi-headed design with different activation functions for the reconstructed feature types.

**Table 9:** Autoencoder Architecture.

Input Dim	Output Dim	Description
**Categorical Branch**		
input_cat_dim	64	Linear + BatchNorm + ReLU
64	32	Linear + BatchNorm + ReLU
**Continuous Branch**		
input_con_dim	64	Linear + BatchNorm + ReLU
64	32	Linear + BatchNorm + ReLU
**Combined Encoder**		
64	30	Linear + BatchNorm + ReLU
30	12	Linear + BatchNorm + ReLU
12	6	Linear + BatchNorm + Sigmoid
**Combined Decoder**		
6	12	Linear + BatchNorm + ReLU
12	30	Linear + BatchNorm + ReLU
**Categorical Decoder Head**		
30	64	Linear + BatchNorm + ReLU
64	input_cat_dim	Linear
**Continuous Decoder Head**		
30	64	Linear + BatchNorm + ReLU
64	input_con_dim	Linear + BatchNorm + Sigmoid

The autoencoder is applied to every time step of the MTS, i.e., an MTS sequence of size ${{\mathbb{R}}}^{\tau \times | V| }$ becomes an *encoded* MTS of size ${{\mathbb{R}}}^{\tau \times | h| }$.

### CNN Classifier

Encoded MTSs are classified using a convolutional neural network. We train $| {\mathcal{T}}\,|$ base classifiers using **D**_train_. Each classification point, $\tau \in {\mathcal{T}}$, has an associated classifier, ${{\mathcal{H}}}_{\tau }$, which is comprised of the autoencoder from the previous section and a CNN classifier.

We experimented with a range of representations. However, the most successful representation involved maintaining the time dimension and treating the ∣*h*∣ features as input channels. We then use 1D convolutional layers to identify patterns in the channels which could be associated with a particular class. Table 10 summarises the classifier architecture used for all base classifiers.

**Table 10:** CNNClassifier Architecture.

Input Channels	Output Channels	Kernel Size	Padding	Stride	Description
6	384	65	32	32	Conv1D + BatchNorm1D + LeakyReLU
384	768	33	16	16	Conv1D + BatchNorm1D + LeakyReLU
768	1536	5	2	2	Conv1D + BatchNorm1D + LeakyReLU
1536	1536	5	2	2	Conv1D + BatchNorm1D + LeakyReLU
—	—	—	—	—	Flatten
12288	3072	—	—	—	FC* 1 + BatchNorm1D + LeakyReLU
3072	1536	—	—	—	FC* 2 + BatchNorm1D + LeakyReLU
1536	12	—	—	—	FC* 3

*Fully Connected Layer.

### Classification Confidence

We adopt an approach from previous literature^63^ which uses a *confidence* value derived from a confidence function, *C*(), and a *confidence threshold*, θ*, to evaluate a classification prediction at each *τ*. If the confidence value of a prediction does not meet the confidence threshold, then the next *τ* in sequence is chosen and another classification is made until the end of the sequence is reached or the confidence level meets or exceeds the threshold. Interestingly, as well as training the base classifiers with **D**_train_ we can also use the same training dataset to generate a confusion matrix. In traditional classification settings, the test dataset is used to generate this confusion matrix to demonstrate the performance of the classifier on unseen data. However, using a confusion matrix derived from the training dataset allows us to quantify the model’s certainty in its predictions, providing an insight into the reliability of classification outcomes. To differentiate these concepts, we will call the confusion matrix derived from the training dataset a *confidence matrix*. By way of an example, say a confidence matrix was generated using a trained classifier in a two class classification problem (${\mathcal{Y}}=\{{y}_{1},{y}_{2}\}$). Subsequently, the model predicts $\widehat{y}={y}_{1}$ for an unseen data point. By referencing values in the confidence matrix, we can start to appreciate how accurate that result is. Moreover, we can generate probabilities from these values if we divide by how many predictions of that label were made using the classifier. Continuing our example, let us say there are 4 instances in our hypothetical training dataset where the classifier predicted *y*_1_: 3 of them are correct and the other is incorrect. Ergo, if the classifier predicts *y*_1_, the probability of a correct classification, $P({y}_{1}| \hat{y}={y}_{1})$, is 75%, while the probability of an incorrect classification is $P({y}_{2}| \hat{y}={y}_{1})$, is 25%. Expanding this concept, we arrive at the *performance* of a classifier, which is defined as:

5 $${r}_{{{\mathcal{H}}}_{\tau }}(y| \widehat{y})=\frac{| | \left\{\{i\,\in \left\{\{1,2,\ldots ,n\}\right\}\,| \,{{\mathcal{H}}}_{\tau }({X}^{(i)})=\hat{y}\,\& \,{y}^{(i)}=y\}\right\}| | }{| | \left\{\{i\,\in \left\{\{1,2,\ldots ,n\}\right\}\,| \,{{\mathcal{H}}}_{\tau }({X}^{(i)})=\hat{y}\}\right\}| | },$$

where *n* is the number of MTS sequences in the training dataset. Equation (5) calculates the probability that *y* is the true class label given that $\widehat{y}$ is predicted for a particular base classifier. In other words, this equation is a formal representation of how we use the confidence matrix to generate the probabilities associated with a specific classification. The denominator of Equation (5) is the set of indices which represent all of the instances within the training dataset where the classifier has predicted $\widehat{y}$, i.e. the sum of the $\widehat{y}$ column within the confidence matrix. Similarly, the numerator of Equation (5) represents the set of indices where $\widehat{y}$ was predicted by the classifier and the actual class label was *y*. While calculating the confidence value, the confidence matrix can be thought of as a lookup table to calculate the classifier performance when a new prediction, $\widehat{y}$, is made using an unseen MTS. In essence, for any given *y*, ${r}_{{\mathcal{H}}}(y| \widehat{y})$ measures the probability that the classifier is wrong if $y\ne \widehat{y}$ or the probability that the classifier is correct if $y=\widehat{y}$.

For a single prediction at *τ*, the performance of a classification result can be used as a confidence value by using the following equation:

6 $${C}_{\tau }(\widehat{y})={r}_{{{\mathcal{H}}}_{\tau }}(\widehat{y}| \widehat{y}).$$

However, more appropriately, a single MTS can be inferred through successive base classifiers at certain classification points, i.e. ${{\mathcal{H}}}_{{\tau }_{1}},{{\mathcal{H}}}_{{\tau }_{2}},{{\mathcal{H}}}_{{\tau }_{3}},\ldots ,{{\mathcal{H}}}_{{\tau }_{T}}$. Therefore, a way of amalgamating multiple confidence values together is needed to consider previous classifications of the MTS. This can be done with the following equation:

7 $${C}_{\tau }({{\mathcal{H}}}_{\tau }(X))=1-\prod _{\tau {\prime} \in {\mathcal{T}},\,\tau {\prime} \le \tau }\left(1-{r}_{{{\mathcal{H}}}_{\tau {\prime} }}({{\mathcal{H}}}_{\tau }(X)| {{\mathcal{H}}}_{\tau {\prime} }(X))\right).$$

To explain further, Equation (7) calculates the confidence of multiple classification results at certain classification points. Note that the *X*s in this equation can be from **D**_validation_ or **D**_test_ but not **D**_train_. As an example of how Equation (7) is used, let us assume that there are two classification points at time steps 5 and 10, ${\mathcal{T}}=\{5,10\}$. With these values in mind, unrolling Equation (7) gives:

8 $${C}_{10}({{\mathcal{H}}}_{10}(X))=1-[(1-{r}_{{{\mathcal{H}}}_{5}}({{\mathcal{H}}}_{10}(X)| {{\mathcal{H}}}_{5}(X)))\,\times (1-{r}_{{{\mathcal{H}}}_{10}}({{\mathcal{H}}}_{10}(X)| {{\mathcal{H}}}_{10}(X)))],$$

which evaluates the confidence of a prediction result, ${{\mathcal{H}}}_{10}(X)$, given that ${{\mathcal{H}}}_{5}(X)$ is predicted in a previous time step.

**Fig. 5:**
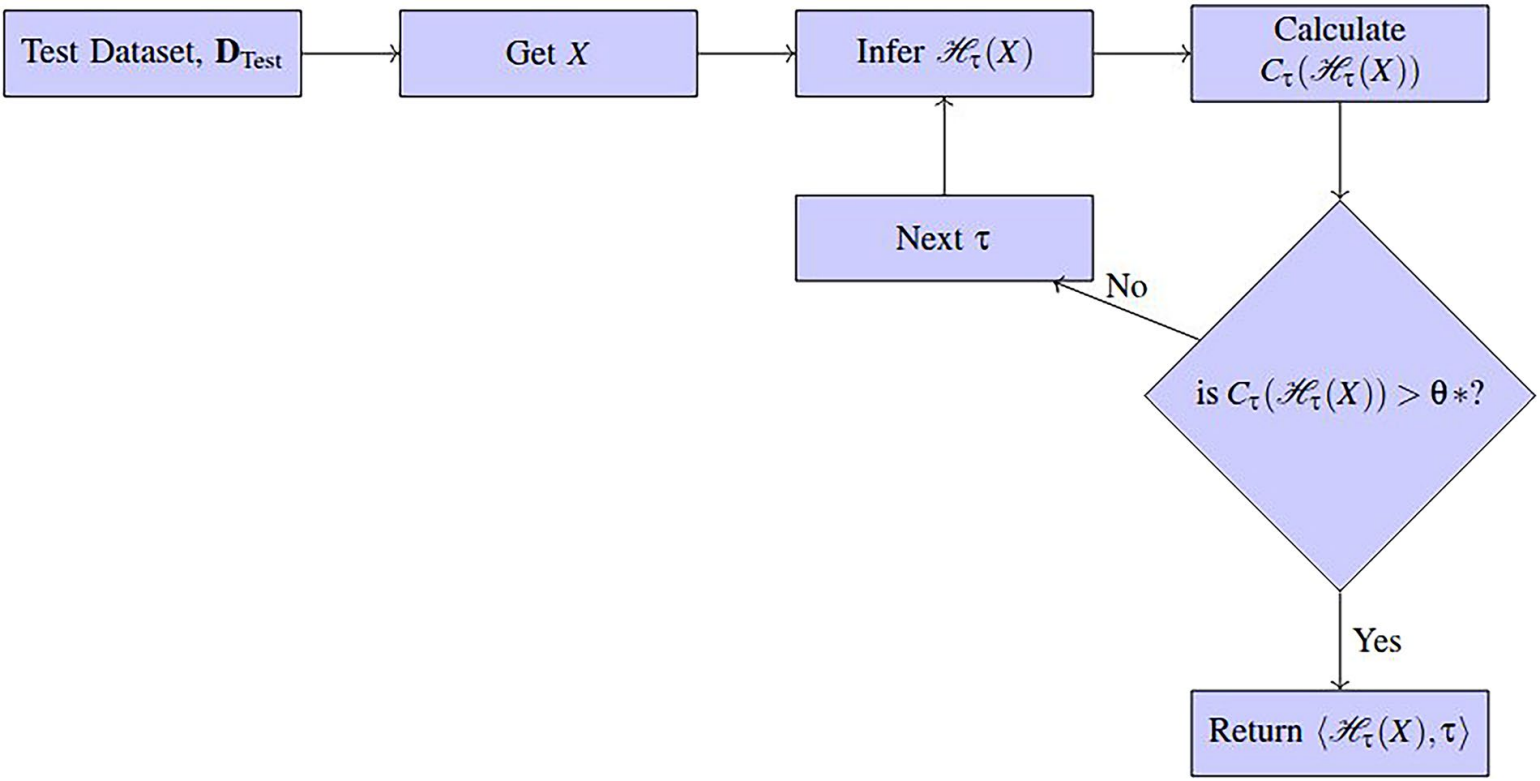
This figure shows the operation of the early classification function (${\mathcal{F}}$) and how the base classifiers interact with the confidence threshold. This figure has been adapted from a previous work^63^.

The early classifier, ${\mathcal{F}}$, uses successive base classifiers as well as a confidence threshold, θ*, to indicate when classification should stop and return the final $\langle \widehat{y},\tau \rangle$ pair. In live operation, the early classifier will start by waiting *τ* time steps to collect incoming data. The resulting *τ* time steps are classified using the appropriate classifier and the confidence is calculated. If the confidence level exceeds θ*, the final classification result and current time step is returned. If the confidence level does not meet this threshold, more time steps are requested and another classification is made at the next *τ* in sequence. This process repeats until a classification and time step is returned. If the confidence never exceeds the confidence threshold, then the result of the last base classifier is used as a classification result. This algorithm is described by Fig. 5.

It is clear that the choice of θ* makes a considerable difference to the Accuracy and Earliness of the early classifier. Therefore, a set of candidates, *θ* ∈ *Θ*, is generated by calculating all the confidence values for every MTS at every $\tau \in {\mathcal{T}}$ in the validation dataset. Duplicate confidence values are removed from the list and the remaining values are sorted in ascending order. The candidate set is then formed by taking the mean of every two consecutive values from the sorted confidence value list. This allows us to create midpoints between known confidence values to use as candidate thresholds.

Each *θ* ∈ *Θ* is tested in the *confidence loss function*:

9 $${L}_{{\rm{conf}}}(\theta )=\alpha (1-Accuracy({\mathcal{F}}\;))+(1-\alpha )Earliness({\mathcal{F}}\;),$$

where *α* is a hyper parameter used to define the trade off between Accuracy and Earliness with the validation dataset used for the calculation of Accuracy and Earliness.

An optimal θ* is chosen which minimises the confidence loss function:

10 $$\theta * =\mathop{{\rm{argmin}}}\limits_{\theta \in \Theta }\left[{L}_{{\rm{conf}}}(\theta )\right].$$

### Experimental Evaluation

To evaluate the validation model, let ${\mathcal{T}}=\{1000,2000,3000,\ldots ,16000\}$). The test dataset, **D**_test_, is used in this section to ensure that none of the training or validation MTSs are used for the experimental evaluation. Other ETSC studies^63,64^ report that an *α* value of 0.9 leads to the best model performance, therefore, we use the same value in this work. For the autoencoder, we define ∣*h*∣ = 6 as the reconstruction loss during training was observed to be minimal. We are cognizant of the fact that these values can be tuned more accurately for increased model performance. However, the purpose of this validation model is to showcase the dataset’s applicability, rather than emphasize any particular performance results. The hyperparameters for the autoencoder and all base classifiers are detailed in Table 11.

**Table 11:** Autoencoder and Base Classifier Hyperparameters.

Model	Hyperparameter	Value
**Autoencoder**	Learning Rate	1e-3
	Number of Epochs	45
	Batch Size	512
**CNN Classifier**	Learning Rate	1e-5
	Number of Epochs	120
	Weight Decay	1e-3
	Batch Size	64

#### Autoencoder and Base Classifier

**Fig. 6:**
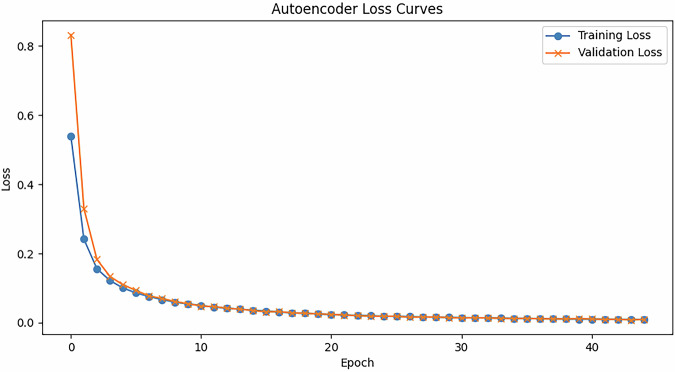
This graph shows the learning curve of the autoencoder.

Figure 6 shows the training curve of the autoencoder. This training curve demonstrates that the autoencoder is able to recreate high dimensional data from a low dimensional embedding space.

**Fig. 7:**
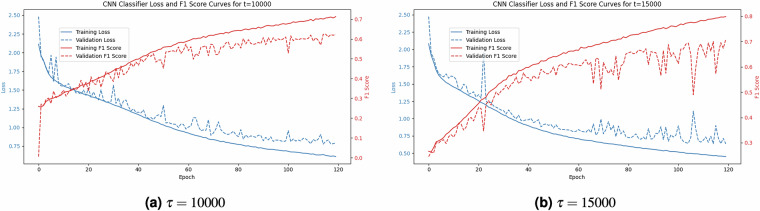
These graphs show the training progress of two base classifiers: ${{\mathcal{H}}}_{\tau =10000}$ and ${{\mathcal{H}}}_{\tau =15000}$. Each graph indicates the F1 score and loss for both the training and validation partition of the FAN-GHETS24^29^ dataset.

In addition, Fig. 7 shows the training graphs of 2 base classifiers, demonstrating how both F1 score increases and loss decreases with increasing epochs in all cases. These learning curves provide evidence that the FAN-GHETS24^29^ dataset includes features which enable the classification of different types and intensities of gray hole attacks. The F1 score is widely used for evaluating classification models, however, Accuracy is more commonly employed for ETSC models^63,65,66^. Thus, we present the performance of the base classifier using the F1 score and the performance of the early classifier using Accuracy.

**Fig. 8:**
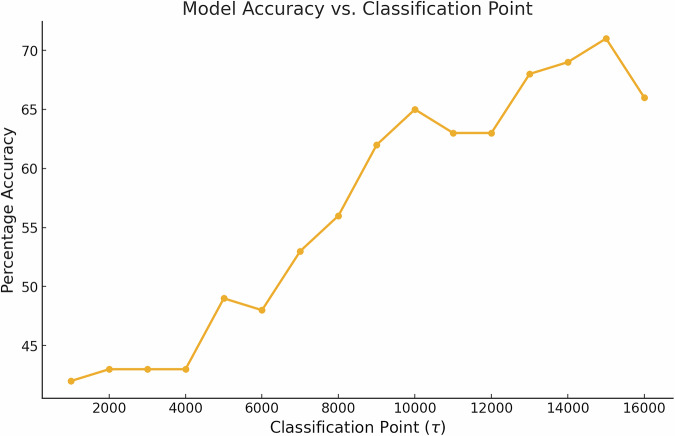
This figure illustrates the relationship between the Accuracy and the classification points which indicate how much of the MTS is used by the base classifiers. As the classification point increases, the Accuracy score generally improves, indicating that classifying with longer MTSs enhances the classifiers’ performance, although the trend exhibits some variability at higher classification points.

**Fig. 9:**
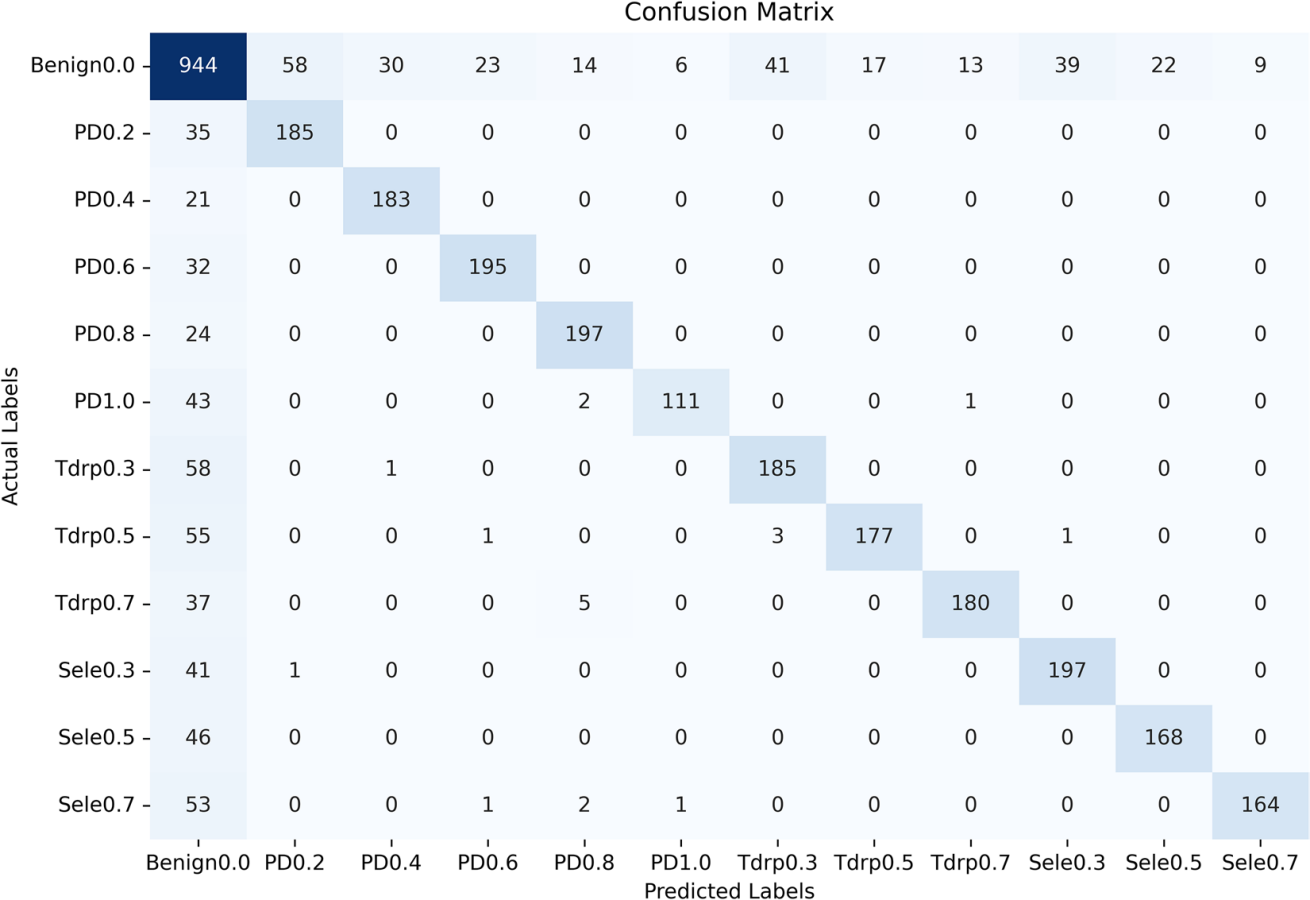
This figure shows the confusion matrix of classifier ${{\mathcal{H}}}_{\tau =15000}$.

Figure 8 shows how the Accuracy varies with *τ* for all base classifiers using the validation dataset. As expected, the longer the MTS, the better the Accuracy of the classifier. In addition, Fig. 9 shows the confusion matrix of classifier ${{\mathcal{H}}}_{t=15000}$ using the validation dataset.

#### Early Time Series Classifier

Table 12 shows the Accuracy and Earliness results of our ETSC model when *α* = 0.7, 0.8 and 0.9. We also show the results of 2 base classifiers, where *τ* = 10000 and *τ* = 15000, and compare these results with our ETSC models. We can conclude that *α* = 0.9 produces the model with the best Earliness and Accuracy score. Consequently, we can compare this result with our base classifiers to justify the utility of ETSC models. The Earliness values for the ETSC models are generated by Equation (3), which calculates the average classification point when using the ETSC model to classify all MTSs in **D**_test_. In contrast, the Earliness of the base classifiers is fixed for every MTS, as there are a fixed number of time steps which the base classifier acts on. Comparing the ETSC results with a base classifier of similar earliness, ${{\mathcal{H}}}_{\tau =10000}$, the early classifier demonstrates an Accuracy improvement of 4%. In addition, while the Accuracy of the other classifier, ${{\mathcal{H}}}_{\tau =15000}$, is 2% higher, the Earliness increases by 32%. This result indicates that while the ETSC model is 2% less Accurate, we can classify sequences much faster than the base classifier. In summary, these results indicate that FAN-GHETS24^29^ is a good candidate for ETSC, as any fixed time sequence classification methods produce notably worse results than the ETSC equivalent.

**Table 12:** This table shows the Accuracy and Earliness of 3 early classifiers each implemented with three different *α* values, *α* = 0.9, *α* = 0.8 and *α* = 0.7.

Classifier	Accuracy	Earliness
Base Classifier (${{\mathcal{H}}}_{\tau =10000}$)	65%	63%
Base Classifier (${{\mathcal{H}}}_{\tau =15000}$)	71%	94%
**Early Classifier** (${\mathcal{F}}$, *α* = 0.9)	**70%**	**62%**
Early Classifier (${\mathcal{F}}$, *α* = 0.8)	69%	62%
Early Classifier (${\mathcal{F}}$, *α* = 0.7)	68%	62%

These early classifiers are compared with the results of 2 base classifiers, ${{\mathcal{H}}}_{\tau =10000}$ and ${{\mathcal{H}}}_{\tau =15000}$. A high Accuracy with a low Earliness indicates a better model.

## Usage Notes

One of the main challenges in using this dataset is the quantity of data captured by each eval node. Much of this data is redundant, therefore, a way of intelligently filtering this data before classification may be beneficial. Alternatively, due to the number of benign MTSs, this dataset could also be treated as an anomaly detection problem before finally classifying the anomaly type (anomalies in this case would refer to types and intensities of grey hole attacks). Our repository, which contains the source code for generating FAN-GHETS24^29^, is designed to support various attack strategies. This allows researchers to explore different threat models, including those not originally included. We also note the class imbalance of FAN-GHETS24^29^ and actively recommend not to balance the classes, as was the case in our validation model. This is because the statistical distribution of real-world occurrences must be faithfully represented in the training dataset^67^. Artificially altering the class distribution risks distorting the model’s predictive capabilities, leading to reduced accuracy.

Ultimately, we recommend using every time step from each sequence when training models with the FAN-GHETS24^29^ dataset. However, similar to previous methods, users may choose to average the metrics over a fixed time window, resulting in a smaller dataset which can be used to train smaller models. If this approach is taken, each resulting sub-sequence may contain a variable number of attack points. To address this, users may wish to define the minimum number of attack points required for a sub-sequence to be classified as a specific intensity or type of GHA.

In general, we recommend the following process to begin working with this dataset:

1. Train a dimensionality reduction model on each data point within the sequence. There are 25 features in total and, in our experience, we found that classification models work best when using an embedding space.
2. Take note of the different types of data in each sequence. We generated a JSON metadata file which contains this data for each sequence. We recommend using this to ascertain how best to manipulate these variables.
3. Use the Pyarrow Python module in combination with Pandas to read the parquet files. In our experiments, it showed noticeable speed increases. Furthermore, we also found it useful to use high memory compute nodes during training to store the full dataset and offload this data to the GPUs as each batch is trained.
4. Train a sequence based classification model. In this work, we used a CNN to validate the dataset, however, we are confident that other model types and architectures can be used. We are very interested to see how other researchers utilise this dataset and the models which are produced as a result.
5. Use an early classification system to determine the optimum classification Accuracy and Earliness from a sequence of classification predictions. We demonstrated the use of a confidence thresholding based system, however, other systems can be used. For example, some systems use the class probabilities from the classifier to determine a set of early stopping rules^16^. We hypothesise that future novel systems could be utilised in combination with our dataset to further improve early classification algorithms in FANETs.

## Data Availability

All source code for the generation and processing of the dataset is available via our public repository: https://git.soton.ac.uk/ch1u20/fan-ghets24/.
